# First molecular evidence and phylogenetic characterization of *Aedes* (*Hulecoeteomyia*) *koreicus* (Diptera: Culicidae) in Slovakia

**DOI:** 10.1007/s00436-026-08628-y

**Published:** 2026-01-28

**Authors:** Katarína Loziaková Peňazziová, Soňa Pivka, Eva Barbušinová, Nasir Ahmad Jalili, Vivien Kiss, Kornélia Kurucz, Tomáš Csank

**Affiliations:** 1 Department of Microbiology and Immunology, University of Veterinary Medicine and Pharmacy in Košice, Komenského 73, Košice, 041 81 Slovakia; 2 Veterinary and Food Institute, Hlinková 1, 043 65 Košice, Slovakia; 3 Institute of Laboratory Medicine, Faculty of Medicine, Slovak Health University, Limbová 12, Bratislava, 833 03 Slovakia; 4 Észak-magyarországi Lógyógyászati Kft, Lévay József utca 1, Miskolc, 3529 Hungary; 5 National Laboratory of Virology, Szentágothai Research Center, University of Pécs, Ifjúság útja 20, Pécs, 7624 Hungary; 6 Institute of Biology, Faculty of Sciences, University of Pécs, Ifjúság útja 6, Pécs, 7624 Hungary

**Keywords:** *Aedes koreicus*, Korean bush mosquito, Slovakia, COI gene, Invasive species, Haplotypes

## Abstract

The invasive mosquito *Aedes* (*Hulecoeteomyia*) *koreicus* (Edwards, 1917), originally native to East Asia, has recently established populations across several European countries. This study provides the first molecular confirmation of *Ae. koreicus* in Slovakia. Adult females were collected during nationwide mosquito surveillance conducted between June and October 2024. Morphological identification was confirmed by sequencing a fragment of the mitochondrial cytochrome oxidase I (COI) gene. Phylogenetic analysis revealed that Slovak *Ae. koreicus* sequences clustered with reference sequences from Italy, Hungary, Belgium, and Germany. BLAST analysis showed 98.4–100% nucleotide identity with European *Ae. koreicus* isolates. Two principal genetic clusters were detected—one related to Hungarian isolates and another to Italian isolates, suggesting multiple introduction pathways or regional spread from established populations in Central Europe. These findings confirm the ongoing expansion of *Ae. koreicus* in Central Europe.

## Background and findings

The increasing spread of invasive *Aedes* mosquitoes across Europe poses a growing public health concern, as several species have been shown to transmit exotic pathogens (ECDC 2025; Ciećkiewicz et al. 2025; Ciocchetta et al. 2018; Jansen et al. 2021). Among these, *Aedes* (*Hulecoeteomyia*) *koreicus* (Edwards 1917), originally endemic to Japan, Korea, northeastern China, and southern parts of Russia (Gutsevich et al. 1971; Tanaka et al. 1979), has shown exceptional adaptability to temperate climates. Following its initial discovery in Belgium in 2008 (Versteirt et al. 2012), *Ae. koreicus* has progressively expanded its European range. Sporadic or spatially restricted populations have been documented in Switzerland (Suter et al. 2015), Slovenia (Kalan et al. 2017), Germany (Werner et al. 2016), Austria (Fuehrer et al. 2020), the Netherlands (Teekema et al. 2022), the Czech Republic (Vojtíšek et al. 2022), and parts of Eastern Europe, such as the Crimean Peninsula and European Russia (Ganushkina et al. 2020; Bezzhonova et al. 2013); whereas the species is widely established in Italy (Gradoni et al. 2021) and Hungary (Kurucz et al. 2016).

This species shows strong ecological adaptability and frequently exploits man-made water-holding structures and other artificial habitats for breeding, especially in urban and suburban environments (Versteirt et al. 2012). Its eggs tolerate low temperatures and dry conditions, which enable survival through winter in temperate zones and facilitate passive dispersal through trade and transport of goods such as used tires (Capelli et al. 2011; Versteirt et al. 2012; Deblauwe et al. 2022; Montarsi et al. 2013; Marini et al. 2019). Unlike some other *Aedes* species, *Ae. koreicus* actively bites both during daytime and nighttime, which enhances its contact with humans (Montarsi et al. 2022; Kim et al. 2003; Tanaka et al. 1979). Moreover, its potential role in the transmission of arboviruses (Ciocchetta et al. 2018; Jansen et al. 2021; Höller et al. 2025) and filarial parasites (Montarsi et al. 2015; KCDC 2007; Kurucz et al. 2018) underscores its significance and emphasizing the importance of extensive monitoring of invasive mosquitoes in Europe.

**Fig. 1:**
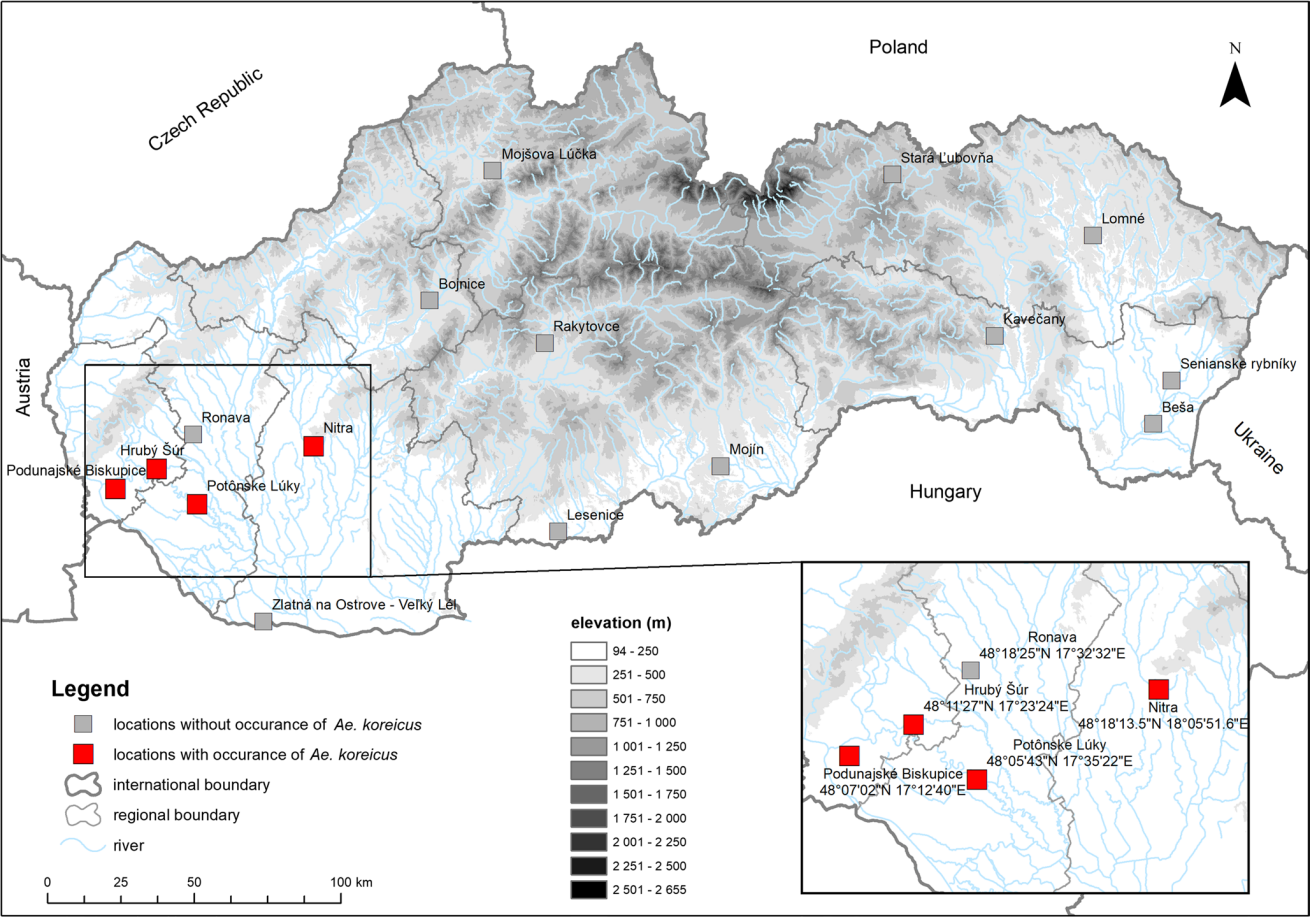
Mosquito trapping localities in Slovakia. Grey squares indicate sites without the presence of *Aedes koreicus* (Ronava, Bojnice, Mojšova Lúčka, Rakytovce, Lesenice, Mojín, Stará Ľubovňa, Kavečany, Lomné, Senianske rybníky, Beša). Red squares indicate sites with confirmed presence of *Ae. koreicus* (Podunajské Biskupice – district Bratislava II, BA; Hrubý Šúr – district Senec, SC; Potônske Lúky – district Dunajská Streda, DS; and Nitra – district Nitra, NR). The inset map in the right corner shows detailed locations where *Ae. koreicus* females were collected, including their corresponding GPS coordinates. Map created by ArcGIS 10.7 software system

The current study provides the first reports on the morphological and molecular confirmation of *Ae. koreicus* detected in Slovakia, in the frame of a nationwide mosquito monitoring in 16 localities across the country (Fig. 1) aimed to establish a One Health surveillance system for emerging pathogens. BG-Sentinel 2 mosquito traps (Biogents AG, Regensburg, Germany) with CO₂ bait were operated continuously from June to mid-October 2024. Trapping nets were collected twice a week, and samples were stored at −20 °C.

During this surveillance programme, more than 40,000 mosquitoes belonging to 23 species were collected. The monitoring was primarily focused on *Culex* mosquitoes, recognised vectors of West Nile virus and Usutu virus, and mosquitoes from other genera were not examined in detail until specimens were observed with morphological characters that did not match any species previously reported from Slovakia. Because *Ae. japonicus* (Theobald, 1901), which is morphologically very similar to *Ae. koreicus*, is already present in Slovakia, only mosquitoes that could be clearly distinguished morphologically were selected for molecular analysis.

**Fig. 2:**
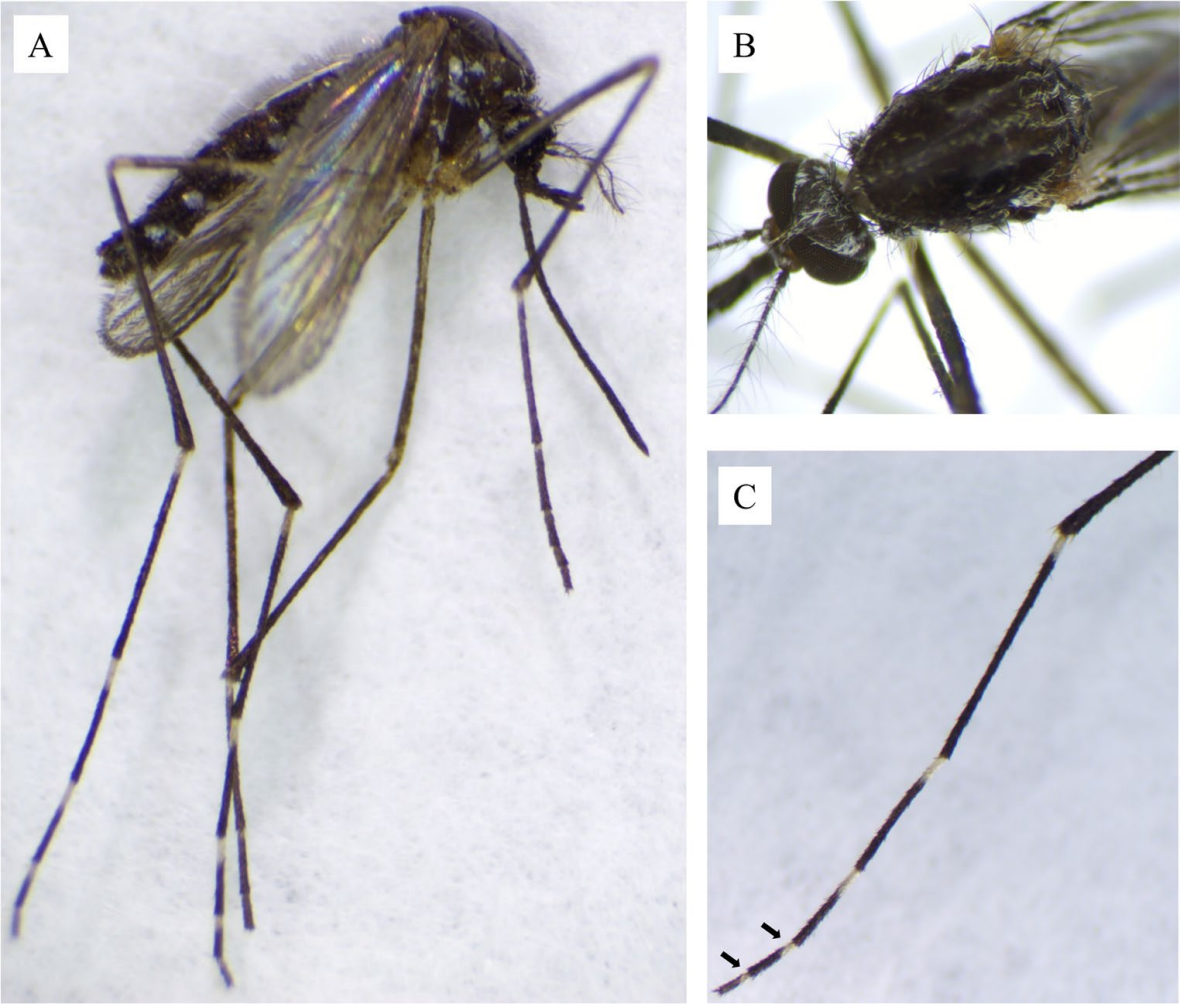
*Aedes koreicus* female: **A **– Whole body, **B** – Occiput, scutum with five stripes of golden lines and scutellum, **C** - Hind tarsomers IV and V with basal pale bands marked by an arrow

A total of six *Ae. koreicus* female specimens (sample IDs: 304.C_BA, 399.C_BA, 992.C_NR, 1068.C_SC, 1201.C_DS, 1284.C_BA) were captured in the western part of Slovakia, mainly in urban areas, and in one case on a horse farm in a rural area (1068.C_SC). All detection sites lie in relative proximity to the Hungarian border, where *Ae. koreicus* is already established. Three of these sites, in the districts Bratislava II, Senec, and Nitra, are also located near major transport hubs and motorway junctions, consistent with passive introduction via human-mediated transport (e.g., the movement of goods or ornamental plants). In contrast, the site in the Dunajská Streda district may reflect a gradual spread from neighbouring established populations. One specimen was collected in June (399.C_BA), one in August (304.C_BA), two in September (1068.C_SC, 1284.C_BA) and two in October (992.C_NR, 1201.C_DS). Mosquitoes were identified based on morphological identification keys (ECDC 2012) under a STMPRO-T Stereo Zoom Microscope (BEL Engineering s.r.l., Monza, Italy) and documented using the Digital Camera EUREKAM 5.0 Plus (BEL Engineering s.r.l., Monza, Italy). The main morphological characteristic confirming the species was the presence of a basal pale band on hind tarsomeres IV and V (Fig. 2).

After morphological identification, DNA was extracted from individual mosquitoes using NucleoSpin^®^ Tissue XS (Macherey-Nagel GmbH & Co., Düren, Germany) according to the manufacturer’s protocol. The COI gene of mitochondrial DNA (mtDNA) was amplified according to Folmer et al. (1994) – F primer (LCO1490, 5′-GGTCAACAAATCATAAAGATATTGG-3′) and Lunt et al. (1996) – R primer (UEA8, 5′-AAAAATGTTGAGGGAAAAATGTTA-3′) using DreamTaqTM Green PCR Master Mix (Thermo Fisher Scientific, Vilnius, Lithuania) and subsequently sequenced by the Sanger method. The obtained reads of partial mtDNA COI genes were de novo assembled and mapped to reference sequences downloaded from GenBank using Geneious 9.1.8 software (Biomatters, Auckland, New Zealand). Consensus sequences of almost 1000 bp were used in Clustal Omega alignment with partial COI gene *Ae. koreicus* sequences downloaded from GenBank. The nucleotide sequences were deposited in GenBank with accession numbers PX472713–PX472718.

Nucleotide BLAST searches showed 98.4–100% nucleotide identity with *Ae. koreicus* COI sequences. Slovak isolates shared 99.2–100% nucleotide (nt) homology. Isolates 304.C_BA, 399.C_BA, and 1284.C_BA from Podunajské Biskupice shared 99.9–100% sequence identity; specimen 399.C_BA had 100% identity with a specimen from Italy (OK668790). Isolate 1201.C_DS from Potônské Lúky shared the highest similarity (99.8%) to both above-mentioned specimens. Isolates 304.C_BA and 1284.C_BA showed the highest similarity (99.9%) to specimens from Italy (OK668790), Germany (OK668774, OK668776), and Hungary (OK668763). Isolates 1068.C_SC from Hrubý Šúr and 992.C_NR from Nitra were almost identical (99.8–99.9%) with the mosquito from Belgium (OK668723). The lowest nt homology (98.4–98.8%) was observed between Slovak isolates and the Slovenian isolate (OK668835).

**Fig. 3:**
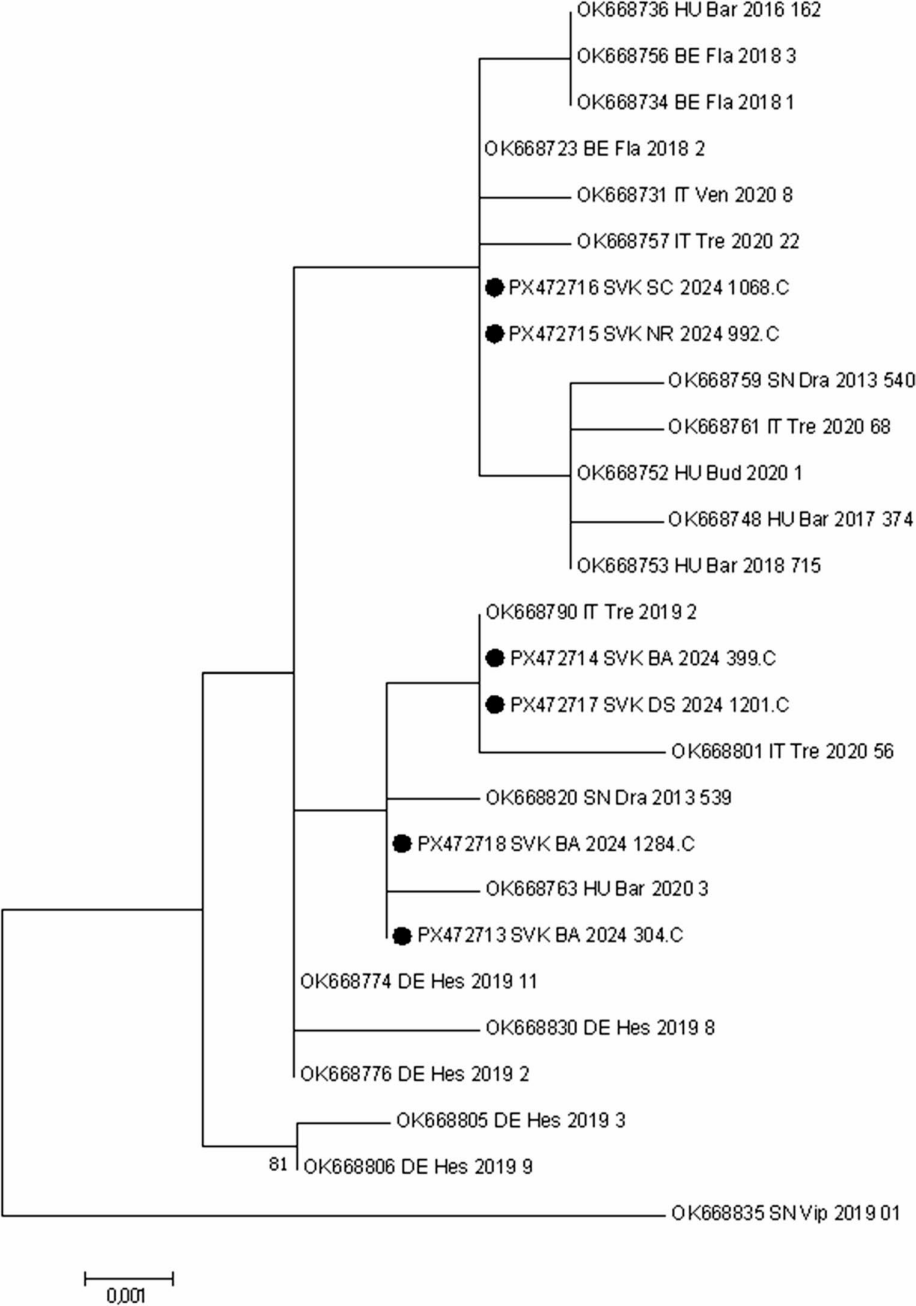
Maximum Likelihood phylogenetic analysis based on the Tamura 3-parameter model (Tamura 1992) was conducted on nucleotide sequences of *Aedes koreicus* isolates from Slovakia using MEGA 7 software (Kumar et al. 2016). The reliability of each tree was estimated by bootstrap analysis of 1000 replicates. A discrete Gamma distribution was used to model evolutionary rate differences among sites (5 categories (+ G, parameter = 0.1000). The percentage above 75% of trees in which the associated taxa clustered together is shown above the branches. The tree is drawn to scale, with branch lengths measured in the number of substitutions per site (below the branches; values below 0.1 are hidden). Slovak sequences are marked with black dots and labelled with accession number, country and region code, year of collection and sample IDs. All other sequences are GenBank reference sequences, labelled with accession number, country and region code, and year of collection. Country codes: SVK – Slovakia, HU – Hungary, IT – Italy, DE – Germany, BE – Belgium and SN – Slovenia

Phylogenetic analysis based on partial mitochondrial sequences revealed that all Slovak *Ae. koreicus* specimens shared a common ancestor with selected references from Belgium, Italy, Germany, and Hungary (Fig. 3). The sequences used for phylogenetic analysis were published by Kurucz et al. (2022), in which 31 haplotypes were described based on pan-European sampling. As demonstrated by Kurucz et al. (2022), *Ae. koreicu*s populations in Europe exhibit notable mitochondrial variability, which can be grouped into five principal COI haplotype clusters. The three most dominant haplotypes were detected across multiple regions, including Belgium, Italy, and Hungary, suggesting dynamic population mixing and recent expansion events across the continent.

The specimens 992.C_NR and 1068.C_SC formed an independent branch positioned next to Belgian (OK668723) and Italian (OK668757, OK668731) isolates. Specimens 1201.C_DS and 399.C_BA clustered together with Italian isolates (OK668801, OK668790). This cluster shared a common ancestor with two other Slovak specimens (1284.C_BA, 304.C_BA) as well as with Slovenian (OK668820) and Hungarian (OK668763) isolates. These results reflect high levels of genetic diversity between and within the examined mosquitoes (Fig. 3). The position of Slovak specimens alongside isolates representing the main COI haplotype clusters suggests that these specimens may belong to the same mitochondrial lineages. This pattern supports the hypothesis of repeated introductions or northward expansion from already established populations in neighbouring countries, consistent with regional dispersal trends observed by Kurucz et al. (2022).

## Conclusion

The presented study reports the first evidence of *Aedes* (*Hulecoeteomyia*) *koreicus* in Slovakia and extends its known distribution in Central Europe. Six female specimens were collected at four sites in western Slovakia, three in urban areas near major transport hubs and motorway junctions and one in a rural area, all in proximity to the Hungarian border. Slovak COI sequences showed close genetic affinity to mosquitoes from Italy, Hungary, Belgium, and Germany. Together, these findings may indicate that local *Ae. koreicus* populations were introduced via human-mediated transport and/or with gradual northward expansion. In the context of ongoing climate change, warmer temperatures and milder winters in Central Europe are likely to support overwintering, population growth and further spread of this cold-tolerant invasive mosquito. These changes may affect native mosquito species composition and increase the risk of transmission of arboviruses and filarial parasites for which *Ae. koreicus* is a potential competent vector. Our findings point to the need for continued surveillance of invasive mosquitoes, genetic monitoring of their populations and systematic assessment of vector-borne disease risks within a One Health framework.

## Data Availability

The data and materials generated during the current study are available from the corresponding author upon reasonable request.
